# *Mycobacterium bovis* Transmission between Cattle and a Farmer in Central Poland

**DOI:** 10.3390/pathogens11101170

**Published:** 2022-10-11

**Authors:** Monika Krajewska-Wędzina, Łukasz Radulski, W. Ray Waters, Anna Didkowska, Anna Zabost, Ewa Augustynowicz-Kopeć, Sylwia Brzezińska, Marcin Weiner

**Affiliations:** 1 Department of Microbiology, National Veterinary Research Institute, 24-100 Puławy, Poland; 2 National Animal Disease Center, Agricultural Research Service, United States Department of Agriculture, Ames, IA 50010, USA; 3 Department of Food Hygiene and Public Health Protection, Institute of Veterinary Medicine, Warsaw University of Life Sciences (SGGW), 02-787 Warsaw, Poland; 4 Department of Microbiology, National Tuberculosis and Lung Diseases Research Institute, 01-138 Warsaw, Poland; 5 Pope John Paul II State School of Higher Education, 21-500 Biała Podlaska, Poland

**Keywords:** *Mycobacterium bovis*, SB0120, transmission, cattle, human, Poland

## Abstract

Introduction: Zoonoses have recently become an increasing public health problem. Zoonoses are estimated to account for 60% of all emerging infectious diseases. One particularly important zoonosis is human tuberculosis, especially tuberculosis due to *Mycobacterium bovis* (*M. bovis*), which is naturally resistant to pyrazinamide (PZA). Material and Methods: The patient had a pulmonary form of tuberculosis accompanied by a cough and fever. At the same time, the disease was also confirmed in 20 out of 25 cattle on the farm. The clinical specimen (sputum) was examined in accordance with the European Union (EU) laboratories’ methodology. Tissue materials from cattle were verified in the National Veterinary Research Institute (NVRI), in the Bovine tuberculosis (BTB) Reference Laboratory, Pulawy, Poland and tested in accordance with the guidelines for the laboratory diagnosis of BTB. Results: All *M. bovis* isolates represented one spoligotype, SB0120. The results of mycobacterial interspersed repetitive unit variable number tandem repeat (MIRU-VNTR) evaluation showed the same genetic pattern. Conclusions: Findings from this study suggest the first confirmed interspecific transmission of *Mycobacterium bovis*, between a farmer and his cattle, in Poland. Present findings support the increasing concern regarding zoonotic TB that has been highlighted elsewhere.

## 1. Introduction

Zoonoses have recently become an increasing public health problem. Zoonoses are estimated to account for 60% of all emerging infectious diseases. One particularly important zoonosis is human tuberculosis (TB) [1,2]. *Mycobacterium* species belonging to the *Mycobacterium tuberculosis* complex (MTBC) are the etiological agents that cause TB in mammals. Genome sequencing has shown that MTBC species have a 99.5% similarity at the nucleotide level and virtually identical 16S rRNA sequences [3]. Human TB is mostly caused by *Mycobacterium tuberculosis*; however, other members of the MTBC (e.g., *Mycobacterium bovis*, *M. africanum,* and *M. caprae*) also infect humans and cause disease [4,5].

Tuberculosis (TB) is now one of the leading causes of death from infectious diseases worldwide, with 10.4 million cases and over 1.5 million deaths in 2019 [6]. It is estimated that, at the beginning of the 20th century, 30% of tuberculosis cases in Europe were caused by the bovine tuberculosis pathogen, *Mycobacterium bovis* [7]. *Mycobacterium bovis* is >99.95% homologous to *M. tuberculosis* [8], largely transmitted to humans by inhalation of infectious droplets from infected cattle or by consumption of contaminated, unpasteurised dairy products [9,10].

Zoonotic tuberculosis was originally considered primarily a disease of children, where the disease involved the cervical lymph nodes (scrofula), the gastrointestinal tract, or the meninges. It is now increasingly recognized that infection in childhood is a precursor to reactivated adult disease, and that many infected children may remain asymptomatic, undiagnosed, and untreated [11,12]. Control and eradication of zoonotic tuberculosis requires early recognition of preclinical infection in animals and prompt removal of all infected animals to eliminate a future source of infection for other animals and humans [13].

The main agent responsible for causing bovine tuberculosis (BTB) in cattle, although it can infect a wide variety of mammals, is *M. bovis* [14,15]. *Mycobacterium bovis* was first described in 1898 by the American microbiologist Theobald Smith [16]. BTB can also be caused by *Mycobacterium caprae*, which was previously identified as a subspecies of *M. tuberculosis* spp. *caprae*. However, when comparing the biochemical and genetic characteristics of this subspecies with *M. tuberculosis*, it was determined that *M. caprae* is a subtype of *M. bovis* [17,18]. In 2003, Aranaz proposed upgrading *M. caprae* to the rank of a species [19]. *M. caprae* is distributed predominantly in Europe [20,21]. According to the available literature and our own research [22], tuberculosis in cattle may also result from *M. tuberculosis* infection [23,24].

Detailed procedures for the suspicion, confirmation, and control of BTB in cattle in Poland, as well as guidelines for performing the tuberculin skin test (TST), are included in the amended *Instruction of the Chief Veterinary Officer*, no. GIWpr-02010-8/2016 [25]. The test protocol includes ante- and post-mortem examinations. The first tests (ante-mortem) comprise the identification of clinical symptoms (usually poorly visible and unrecorded in Poland for several dozen years) and the results of the TST. Tuberculin can be used in Poland; however, it must be included in the current list of pharmaceuticals authorized for marketing and undergo serial quality assurance testing in accordance with the requirements of the World Organization for Animal Health (OIE) and the current edition of the *European Pharmacopoeia.* In Poland, 20% of cattle herds are tested each year via TST, which means that the entire cattle population in Poland is tested at 5-year intervals. If a TST result is positive, the cattle are euthanized, and post-mortem tests are performed in the National Reference Laboratory of Bovine Tuberculosis in Poland. Since 2009, Poland has met the conditions set out by the Regulation 2020/689 of 17 December 2019 supplementing Regulation (EU) 2016/429 of the European Parliament and of the Council and is regarded as being officially free from BTB (OTF) [26]. Despite this, tuberculosis in cattle is still recorded in Poland. Over the past 5 years, the number of outbreaks has fluctuated between 9 and 33. In 2018, there were 9 confirmed outbreaks of BTB, with the most significant disclosing 127 reactors in a single herd (unpublished data). The number of BTB cases found in abattoirs has also increased in recent years. This is a disturbing finding given that infected animals were detected during post-mortem examination at a slaughterhouse, rather than via live animal surveillance using the single intradermal comparative cervical tuberculin test (SICCT). This finding is most likely related to the fact that, since Poland obtained OTF, the time interval of BTB diagnostic testing has increased. Even 12 years ago, the entire cattle population was tested on a 3-year interval, not every 5 years, as it is now.

## 2. Materials and Methods

### 2.1. Human

In February 2016, a 32-year-old male agricultural worker (i.e., a cattle breeder) from central Poland was diagnosed with pulmonary tuberculosis. The clinical specimen (sputum) was processed with N-acetyl-l-cysteine and sodium hydroxide [NALC–NaOH, (Sigma-Aldrich, St. Louis, MO, USA)], followed by centrifugation (Hermle, Wehingen, Germany), and then resuspended in 3 mL of phosphate buffered saline (Chempur, Piekary Śląskie) and subjected to microscopy with Ziehl–Neelsen staining (Becton Dickinson Diagnostic Systems, Sparks, MD, USA) and cultivation on both solid media [egg-based Löwenstein–Jensen medium and Stonebrink’s medium (Grasso, Jabłonowo, Poland)] and a liquid medium (BACTEC MGIT 960 culture; BD Microbiology Systems, Maryland, USA). Identification of the *M. tuberculosis* species was performed using the niacin test (Becton Dickinson Diagnostic Systems, Sparks, MD, USA) and molecular method (Hain Lifescience, Nehren, Germany). Drug resistance/susceptibility of the *M. tuberculosis* complex strain was determined using solid media (Grasso, Jabłonowo, Poland) in accordance with the methodology applied in EU laboratories [27].

### 2.2. Animals

The most recent SICCT for this herd had been applied in 2011, and there were no reactors. Worth mentioning, cattle were added to the herd between 2011 and 2016, but details on the animals’ movements are unknown. In March 2016, the Veterinary Inspection Service received information from the Sanitary and Epidemiological Station about the occurrence of TB in a person on the farm. Two weeks later, a comparative intradermal tuberculin test (CIDT) was performed using avian (Avitubal 28,000 IU/mL; Bioveta a.s., Czech Republic) and bovine tuberculin (Bovitubal 28,000 IU/mL; Bioveta a.s., Ivanovice na Hané, Czech Republic). A total of 25 head of cattle were tested. The cattle were all females, ranging from 2 to 8 years of age. The CIDT was read 72 h later, using a version for a herd in which tuberculosis has already been diagnosed. This is a more stringent test used in accordance with Polish veterinary law [25]. All 25 cows on the farm were humanely euthanized. The lymph nodes—retropharyngeal, mandibular, bronchial, mediastinal, mesenteric, and supramammary—were sent to the National Veterinary Research Institute (NVRI), Puławy, Poland. Tissue materials were very carefully sliced and shredded, the different lymph nodes were pooled, and a single sample was processed for culture. Finally, each sample consisted of slices of individual lymph nodes from each cow. A total of 21 samples from dissected tissues obtained were homogenized in 5% oxalic acid. The first step of the homogenate was incubated at 37 °C (±2 °C) for 10 min; then, the homogenate was centrifuged. After that, it was flushed twice with 0.85% NaCl to obtain a culture sediment. Based on official BTB diagnostic guidelines, a culture on Stonebrink’s solid medium was performed. Isolates were then evaluated by microbiological examination using the NVRI BTB Reference Laboratory, as previously described by Lipiec et al. [15]. The reagents used for these examinations were prepared in the Media Department (NVRI, Puławy, Poland).

### 2.3. Genetic Analysis

Genetic analysis was performed to compare the isolates from the farmer and his cattle. This portion of the study was completed in the National Tuberculosis and Lung Diseases Research Institute, Warsaw, Poland. The MTBC GenoType Test^®^ (Hain Lifescience, Nehren, Germany) was used for the identification of MTBC from the farmer and his cattle (21 MTBC strains). Genotyping based on spoligotyping (Ocimum Biosolutions, Hyderabad, India) was conducted according to the standardized protocol described previously [28]. The spoligotype patterns were compared to those of strains registered in the SpolDB4 database [29]. Spoligotypes were assigned according to international spoligotype nomenclature [30]. The 15-MIRU-VNTR was conducted manually for the 15 loci, according to Rodriguez [31]. The following loci were analysed: MIRU-4, MIRU-10, MIRU-16, MIRU-26, MIRU-31, MIRU-40, VNTR 424, VNTR 577, VNTR 2165, VNTR 2401, VNTR 3690, VNTR 4165, VNTR 2163b, VNTR 1955, and VNTR 4052. For PCR, the Taq DNA polymerase kit (EurX, Gdansk, Poland) was used. For electrophoresis, a 2% agarose gel (Sigma-Aldrich, St. Louis, MO, USA) was used with the H37Rv strain as a positive control. The allele copy numbers were added manually to a Microsoft Excel spreadsheet for analysis.

## 3. Results

### 3.1. Human Case

**Figure 1:**
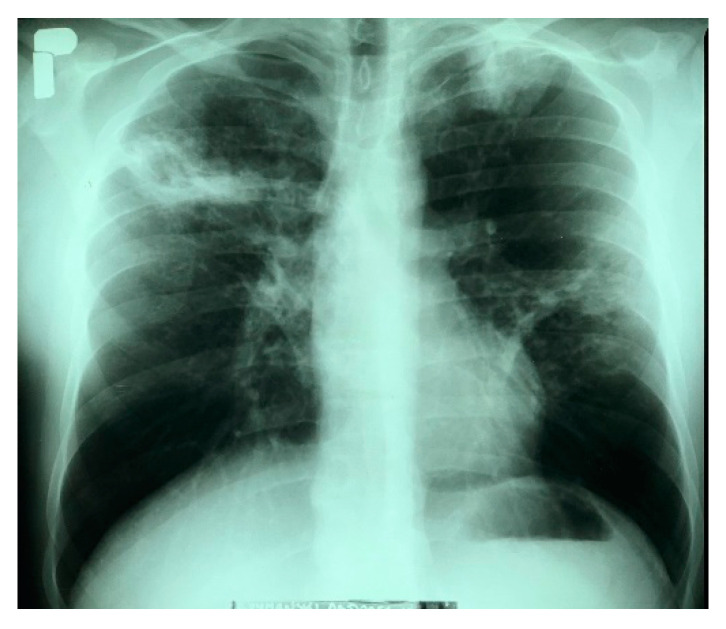
The X-ray image taken before the start of antituberculosis treatment.

The patient was admitted to the Provincial Complex of Healthcare Institutions, Lung Disease Treatment and Rehabilitation Center in Łódź, II Department of Lung Diseases, (Tuszyn, Poland) because of profuse haemoptysis, which had occurred for the first time in his life. During the anamnesis, the patient complained of a persistent cough that had first occurred 6 months prior. For 2 months, the cough was accompanied by hoarseness and a high fever (slightly above 39 °C), which intensified mainly in the evenings. At the same time, the patient had night sweats. The man suffered from malaise, a lack of appetite, and a weight loss of 6 kg. He did not report any further symptoms, had not received chest radiographs for many years, and there was no history of tuberculosis within his immediate family. The patient disclosed that he had smoked cigarettes since the age of 20. The X-ray image of the lungs revealed an infiltrative lesion in the upper field of the right lung, with a cavity of approximately 35 × 30 mm, an infiltrative lesion in the left apex, and nodular fibrous lesions in the middle field of the left lung. The left diaphragm and diaphragm-costal angles were without pathological changes. The heart silhouette was within normal limits (Figure 1).

**Figure 2:**
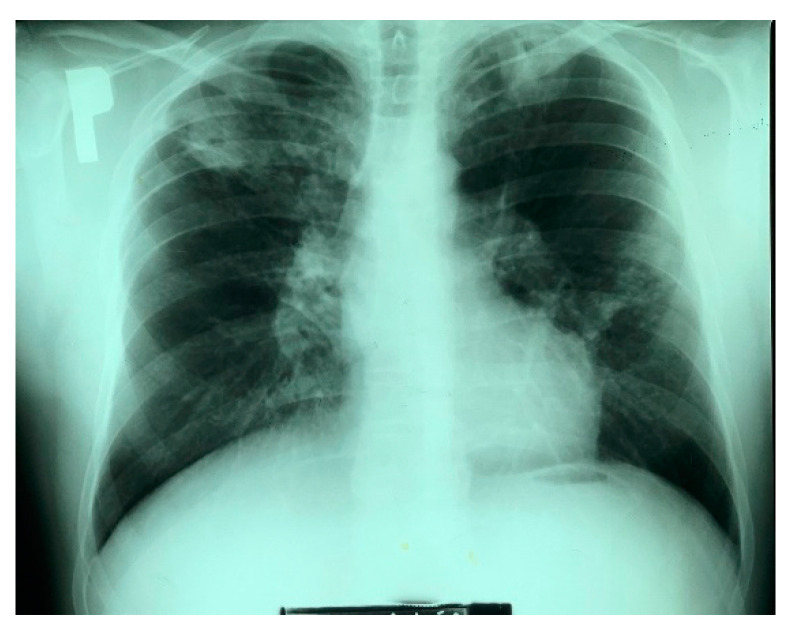
The X-ray image taken after 6 weeks of antituberculosis treatment.

Using the direct sputum test for TB, acid-fast mycobacteria were not detected; however, 3 weeks of incubation on Stonebrink’s medium resulted in the growth of *Mycobacterium*. Initial identification in the hospital laboratory confirmed that the isolate belonged to the *Mycobacterium tuberculosis* complex. Molecular identification (MTBC GenoType Test ^®^, Hain Lifescience, Nehren, Germany) of the isolate revealed that it was *Mycobacterium bovis*. Antibiotic resistance testing demonstrated sensitivity to streptomycin (SM), isoniazid (INH), rifampicin (RMP), ethambutol (EMB) and resistance to pyrazinamide (PZA). On the basis of the radiograph of the lungs and the results of the sputum evaluation, the patient was diagnosed with active TB. An ophthalmological consultation showed no contraindications to EMB treatment. The antituberculosis treatment was implemented: RMP 0.6 g; PZA 1.5 g; and EMB 4 capsules. The treatment additionally included amikacin, cyclonamine, aminocaproic acid, codeine, 10% NaCl, dexamethasone, omeprazole, and a preparation for protecting the liver (Silimarol ^®^, Herbapol, Poznań, Poland). The patient tolerated the treatment well. There were episodes of haemoptysis during hospitalization, yet the patient’s fever resolved with antibiotic treatment without recurrence. After 6 weeks of treatment, there was clinical and partial radiological improvement (Figure 2). The patient was discharged with recommendations for further treatment in a pulmonary clinic. The entire antituberculosis treatment lasted 6 months.

### 3.2. Herd History

The animals showed no clinical signs of tuberculosis. CIDT revealed 21 reactors. The results of necropsy of individual tissues are summarized in Table 1. Among the 21 cows tested, tuberculous findings in the lymph nodes were observed in 20 animals, and in these animals, BTB was confirmed. The isolates, which were obtained from 20 animals, were identified as *Mycobacterium bovis*.

**Table 1:** Lymph nodes from 21 reactor cattle—results of pathology examination of individual tissues.

No.	Cattle Numbers as Marked in the Laboratory	Macroscopic Findings					
		Mandibular	Retropharyngeal	Bronchial	Mediastinal	Mesenteric	Supramammary
1.	T-135, T-145, T-140, T-150, T-151, T-154, T-153	enlarged and haemorrhagic	Firm, white nodules	caseous tubercles	caseous tubercles	Haemorrhagic and/or enlarged	enlarged and haemorrhagic
2.	T-136	clear	enlarged and haemorrhagic	caseous tubercles	caseous tubercles	haemorrhagic	enlarged and haemorrhagic
3.	T-137	whitish lumps	enlarged and haemorrhagic with fibrous, pale-yellow lesions (Figure 3)	caseous tubercles	caseous tubercles	haemorrhagic	enlarged and haemorrhagic
4.	T-138	enlarged and haemorrhagic	enlarged and haemorrhagic	caseous tubercles (Figure 4)	caseous tubercles	haemorrhagic	enlarged and haemorrhagic
5.	T-139	clear	clear	clear	clear	clear	clear
6.	T-141, T-143, T-152	enlarged and haemorrhagic	clear	caseous tubercles	caseous tubercles	haemorrhagic and/or enlarged	enlarged and haemorrhagic
7.	T-142, T-146	clear	clear	caseous tubercles	caseous tubercles	clear	enlarged and haemorrhagic
8.	T-144	whitish lumps	clear	caseous tubercles	caseous tubercles	clear	enlarged and haemorrhagic
9.	T-147, T-148	clear	firm, white nodules	caseous tubercles	caseous tubercles	clear	enlarged and haemorrhagic
10.	T-149	clear	clear	enlarged and haemorrhagic	clear	clear	clear
11.	T-155	enlarged and haemorrhagic	clear	caseous tubercles	caseous tubercles	clear	enlarged and haemorrhagic

### 3.3. Genetic Analysis

Isolates from the farmer and each of the 20 infected cattle formed a single cluster: *M. bovis* spoligotype pattern, SB0120 ^1^ assigned by www.Mbovis.org (accessed on 21 August 2022) and Bov_1 482 ^2^—assigned by SITIVIT. The MIRU-VNTR results of the 21 strains presented 1 pattern 422432155421434 (Table 2).

## 4. Discussion

**Figure 5:**
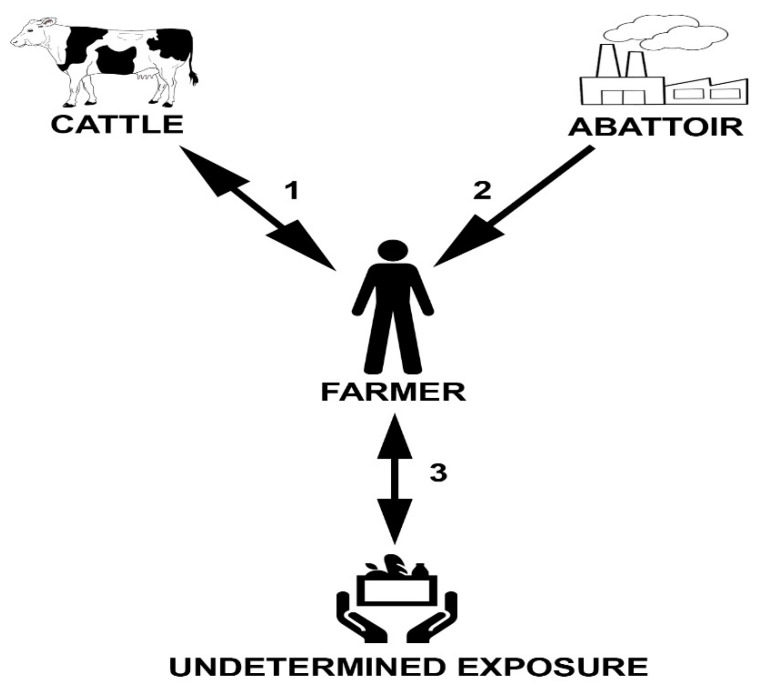
Possible pathways of *M. bovis* transmission, which include: (**1**) farmer infected while working at abattoir, transmitting *M. bovis* to cattle on his farm; (**2**) farmer infected by exposure to *M. bovis*-infected cattle on his farm; (**3**) farmer infected with *M. bovis* by undetermined exposure (e.g., environmental, ingestion of M. bovis-contaminated dairy products or food, or human-to-human transmission) with transmission to his cattle.

Findings from this study suggest the first confirmed interspecific transmission of *Mycobacterium bovis*, between a farmer and his cattle, in Poland. It may be speculated about three possible pathways of pathogen transmission, which include: (1) the farmer was infected while working at a slaughterhouse, transmitting *M. bovis* to the cattle on his farm; (2) the farmer was infected by exposure to *M. bovis*-infected cattle on his farm; and (3) the farmer was infected with *M. bovis* by an undetermined exposure (e.g., environmental, ingestion of *M. bovis*-contaminated dairy products or food, or human-to-human transmission) with transmission to his cattle (Figure 5). The farmer who fell ill with *M. bovis* tuberculosis worked for 8 years in a slaughterhouse, where cattle BTB reactors were also killed. It may be speculated that he became infected with BTB from exposure to tuberculous animal(s) within the slaughterhouse without following the rules of hygiene and safety while working with infectious material. Despite the fact that he worked in a protective apron and gloves, he did not take off his dirty protective gloves during breaks when he went outside to smoke a cigarette. Then, he potentially transmitted *M. bovis* to his cattle on the farm and fell ill with active BTB himself.

The clinical symptoms of bovine tuberculosis most often include emaciation, lack of appetite, coughing, and fluctuating fever [32]. However, BTB can spread between animals in a herd without clinical signs until the disease has been established within the herd [33]. Menin et al. proved that asymptomatic disease can develop in up to 92% of infections. A similar situation took place with regard to cattle in the analysed case, in which no individual showed clinical symptoms of the disease.

In human tuberculosis caused by *M. bovis*, clinical signs of the disease can be observed much more frequently. In people, symptoms of TB disease caused by *M. bovis* are similar to the symptoms of TB caused by *M. tuberculosis*. This can include fever, night sweats, and weight loss. Other symptoms might occur depending on the part of the body affected by the disease [34]. In the course of tuberculosis in the described case, the man experienced night sweats, fever, and cough, which are the same as those in the case described by Lombard et al. [35]. The subject of this research was a man who worked in a dairy from January to March 2015 in the USA. The case-patient presented in the emergency room with night sweats, cough, and fever. Sputum smears were positive for acid-fast bacteria, and a nucleic acid amplification test (NAAT) was positive for MTBC. The culture revealed the case-patient was infected with *M. bovis*. It should also be noted that the profession of both men was closely related to contact with animals. Moreover, both in the case of the patient from the USA and in the present case, the disease took the form of cavitary pulmonary disease, which is also characteristic of tuberculosis caused by *M. tuberculosis* [36]. This fact confirms that the course of the disease caused by these two different pathogens is very similar.

Considering the fact that *M. bovis* is naturally resistant to the PZA antibiotic, which is used in the treatment of tuberculosis caused by *M. tuberculosis*, efficient species identification of the pathogen is needed, also allowing for the identification of individual strains of a given mycobacterium species [37]. Species identification within the MTBC is of particular importance for epidemiological purposes, especially to elucidate zoonotic contributions. Molecular typing of *Mycobacterium* strains allows for the tracing of the chain of transmission in the environment and can be used to follow independent epidemics. Undoubtedly, the diagnostic techniques that are increasingly used in laboratory practice include spoligotyping and MIRU-VNTR, which were also used in the presented case [38].

The results of spoligotyping and MIRU indicated a common source of infection. All *M. bovis* isolates represented one spoligotype, SB0120, which is widespread in Europe and a common spoligotype circulating on other continents [39,40,41]. Branger and co-authors have proposed a new clonal complex, termed European 3, that is dominated by SB0120-spoligotype strains. It was recently described as “clonal group I” or “BCG-like” [42,43,44], based on whole-genome single-nucleotide polymorphism (SNP) analysis of 50% French MTBC strains isolated from cattle or wildlife [39].

## 5. Conclusions

Present findings support the increasing concern of zoonotic TB that have been high-lighted elsewhere. Due to diagnostic and financial limitations concerning the identification of *M. bovis* and *M. caprae* in human medical laboratories, the number of bovine tuberculosis cases in the Polish population does not reflect the actual number of cases. Although the location of BTB outbreaks in Poland and possible routes of BTB transmission are known, the epidemiological map of this zoonotic disease in Poland may be underestimated.

## Figures and Tables

**Figure 3:**
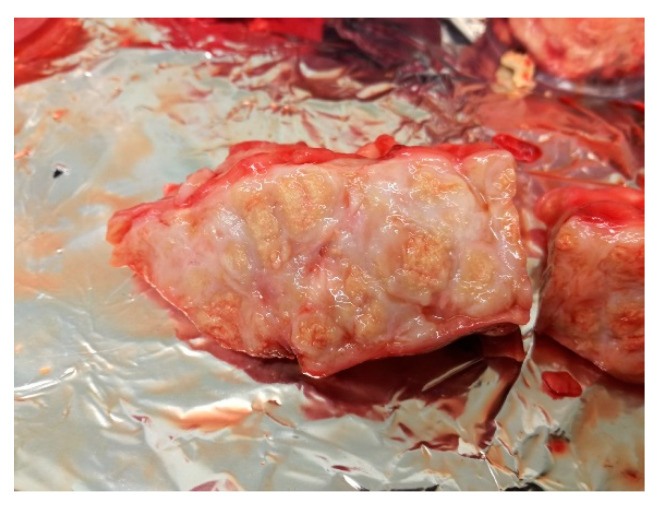
Haemorrhagic, enlarged retropharyngeal lymph node with fibrous, pale-yellow lesions.

**Figure 4:**
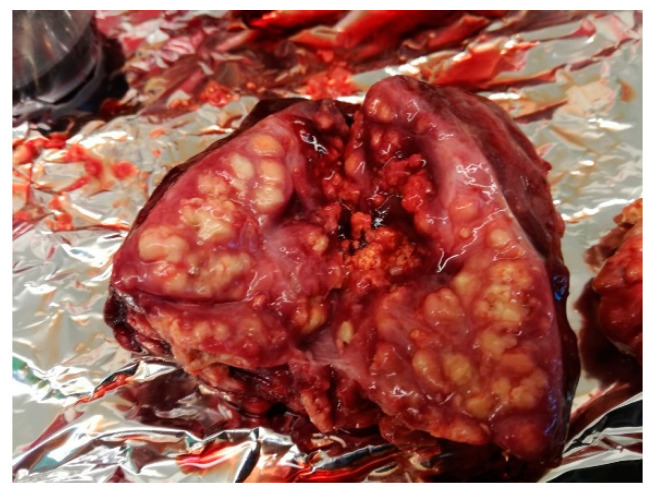
Haemorrhagic, enlarged bronchial lymph node with caseous tubercles.

**Table 2:** Identification of *Mycobacterium bovis* isolates.

No.	Strain Number	Spoligotyping		MIRU Pattern (Number of Strains Possessing This MIRU Pattern)
		Assigned by www.Mbovis.org ^1^ Assigned by SITIVIT ^2^		
1	Strain isolated from the farmer	SB0112 ^1^	*Bov_1 482* ^2^ 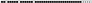	422432155421434 (1)
2	bovine strains T-135-155	SB0112 ^1^	*Bov_1 482* ^2^	422432155421434 (20)

## Data Availability

Data are available in the Department of Microbiology, National Veterinary Research Institute, Puławy, Poland.
